# Thyroxin and calcitonin secretion into thyroid venous blood is regulated by pharyngeal mechanical stimulation in anesthetized rats

**DOI:** 10.1007/s12576-019-00691-8

**Published:** 2019-07-03

**Authors:** Kaori Iimura, Harue Suzuki, Harumi Hotta

**Affiliations:** Department of Autonomic Neuroscience, Tokyo Metropolitan Institute of Gerontology, 35-2 Sakaecho, Itabashi-ku, Tokyo, 173-0015 Japan

**Keywords:** Thyroxin, Calcitonin, Superior laryngeal nerve, Pharynx, Mechanical stimulation, Reflex

## Abstract

The effects of the pharyngeal non-noxious mechanical stimulation on the secretion of immunoreactive thyroxin (iT4), immunoreactive calcitonin (iCT), and immunoreactive parathyroid hormone (iPTH) into thyroid venous blood were examined in anesthetized rats. Secretion rates of iT4, iCT, and iPTH were calculated from their concentration in thyroid venous plasma and the plasma flow rate. A mechanical stimulation was delivered to the pharynx by a rubber balloon placed on the tongue that was intermittently pushed into the pharyngeal cavity. Pharyngeal stimulation increased iT4 and iCT secretion, but iPTH secretion was unchanged. The secretion responses were abolished by transecting the superior laryngeal nerves (SLNs) bilaterally. The activities of the thyroid parasympathetic efferent nerves and the afferent nerves in the SLN increased significantly during pharyngeal stimulation. These results indicate that pharyngeal mechanical stimulation promotes thyroxin and calcitonin secretion from the thyroid gland by a reflex increase in SLN parasympathetic efferent activity, triggered by excitation of SLN mechanoreceptive afferents.

## Introduction

The autonomic nerve fibers’ distribution to widespread visceral organs contributes to the regulation of various physiological functions. Endocrine functions are not exceptional. These include catecholamine secretion from the adrenal medulla and estradiol secretion from the ovary, leading to changes depending on the efferent activity of the sympathetic adrenal or ovarian branches, respectively [1–3]. The thyroid gland, a large endocrine organ attached to the larynx and the upper part of the trachea, receives innervation from sympathetic and parasympathetic thyroid branches derived from the cervical sympathetic trunks (CSTs) and the superior laryngeal nerve (SLN), respectively [4, 5].

Recently, we have shown, by stimulating at a supramaximal intensity the cut peripheral portion of either CSTs or SLNs, that the sympathetic (inhibitory effects) and parasympathetic (excitatory effects) efferent fibers antagonistically regulate the secretion of immunoreactive thyroxin (iT4), immunoreactive triiodothyronine (iT3), and immunoreactive calcitonin (iCT) from the thyroid gland, whereas the sympathetic nerve promotes the secretion of immunoreactive parathyroid hormone (iPTH) from the parathyroid gland [6]. Furthermore, when intact SLNs were stimulated at low-current intensity to selectively excite thick myelinated fibers, secretion of iT3, iT4, and iCT increased, but secretion of iPTH did not change; these responses were similar to those produced by stimulation of cut peripheral SLNs [6]. The majority of myelinated fibers in the SLN are afferent fibers [7], and pre- and postganglionic autonomic efferent fibers are unmyelinated in rats [7–9]. These findings led us to hypothesize that excitation of sensory afferents in SLN may produce a reflex increase in the efferent activity of the SLN parasympathetic thyroid branches to promote hormonal secretion into thyroid venous blood.

The hormonal secretions from the aforementioned adrenal medulla and the ovary were shown to be regulated reflexively by natural somatosensory stimuli via changes in autonomic nerve activity [1, 10–14]. In anesthetized rats, the innocuous mechanical stimulation of the skin produces a reflex decrease in adrenal sympathetic efferent nerve activity, leading to a decrease in catecholamine secretion from the adrenal medulla [1, 10]. On the other hand, the nociceptive mechanical stimulation of the skin produces a reflex increase in ovarian sympathetic nerve activity, leading to a decrease in estradiol secretion from the ovary [13, 14]. In this manner, natural somatosensory stimuli can influence various autonomic functions as specific reflex responses, even after emotional factors are eliminated by anesthesia [1]. Therefore, we can predict that the thyroid function may be regulated by specific natural sensory stimuli conducted to the brain via SLN afferents, due to a reflex change in thyroid parasympathetic efferent nerve activity. However, the natural sensory stimuli that trigger such a neurogenic reflex in the thyroid are unknown and remain to be identified.

SLN contains myelinated afferent fibers that conduct information from the mechanoreceptors in the pharynx [15–17], and the swallowing reflex is induced by stimulation of SLN myelinated afferents [18–20]. The epithelia of the pharyngeal mucosa have an even richer innervation than that of the densely innervated perioral skin and oral cavity [21]. We expected that the mechanical stimulation of the pharynx would produce a reflex response of hormonal secretion from the thyroid gland via the SLN. This study aimed to clarify whether pharyngeal mechanical stimulation promotes iT4 and iCT secretion from the thyroid gland, without changes in iPTH secretion from the parathyroid gland, in anesthetized rats, and if so, whether the SLN is involved in that reflex using afferent and efferent pathways.

## Methods

The experiments were performed in 17 male Sprague–Dawley rats (3–6 months of age, 440–700 g, purchased from Japan SLC, Inc., Shizuoka, Japan). The animals were housed at a constant ambient temperature of 22 ± 1 °C under artificial light (between 0800 and 2000 h) and were fed laboratory chow (CRF-1 LID6, Oriental Yeast Co., Tokyo, Japan) and water ad libitum. The study was conducted with the approval of and in accordance with the guidelines for animal experimentation prepared by the Animal Care and Use Committee of Tokyo Metropolitan Institute of Gerontology.

Animals were used for three different experiments, i.e., blood sampling (*n* = 4), afferent nerve activity recording (*n* = 5), and efferent nerve activity recording (SLN: *n* = 6; cervical sympathetic nerve: *n* = 3). Animals were anesthetized with urethane (1.1 g/kg, i.p.). The trachea was cannulated for ventilation using a respirator (SN-480-7; Shinano Seisakusho, Tokyo, Japan). The end-tidal CO_2_ concentration was maintained at 3.5–4.5% by monitoring with a gas analyzer (Capnostream 20p, Oridion Medical, Jerusalem, Israel). The femoral vein and artery of one hindleg were cannulated for infusing solutions and measuring systemic arterial blood pressure, respectively. The rectal temperature was maintained at 37–38 °C (set at 37 °C during surgery and then increased to 37.5 °C during data collection) using a direct current heating pad and an infrared lamp (ATB-1100, Nihon Kohden, Tokyo, Japan). During experiments, an additional dose of urethane (10–20% of initial dose) was administered intravenously if necessary to keep the level of anesthesia needed to avoid withdrawal reflex and preserve blood pressure stability.

### Mechanical stimulation of the pharynx

**Fig. 1:**
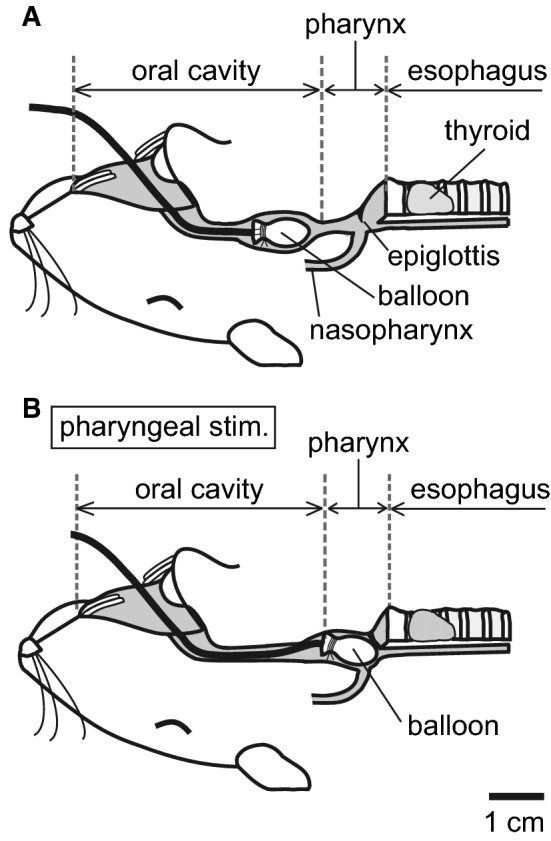
Schematic diagram showing the method of pharyngeal stimulation used in this study, which illustrates the balloon position before (**a**) and during (**b**) stimulation. **a** Original position of the balloon. The balloon inserted from the mouth was placed in the oral cavity approximately 3 cm caudal from the rostral end of the mandible. **b** Balloon position during pharyngeal stimulation. The balloon was pushed, manually via the catheter connected, into the pharyngeal cavity to apply a mechanical stimulation of the pharyngeal areas, including the epiglottis

Mechanical pharyngeal stimulation was delivered using a deflated thin rubber balloon (length: approx. 7 mm, made of a condom tip) attached to a polyethylene catheter (outer diameter: 0.8 mm). The balloon was placed on the tongue at approximately 1 cm distant from the pharynx (Fig. 1a). For stimulation, the balloon was pushed into the pharyngeal cavity (Fig. 1b), usually for a period of 1 s, and then pulled back to the original position (Fig. 1a). The intermittent stimulation of the pharynx was repeated once every 10 s, i.e., at a frequency of 0.1 Hz. We confirmed the position of the balloon reached the pharynx by inducing swallowing reflex.

Intermittent pharyngeal stimulation was performed for 6–14 min during blood sampling experiments until the blood volume collected reached approximately 250 μl. The intermittent pharyngeal stimulation was repeated two to three times in each rat, at an interval of at least 1 h, based on preliminary studies showing that hormonal secretion was reproducible under the same conditions after 1 h.

### Collection of thyroid venous blood plasma and determination of secretion rate of hormones

Thyroid venous blood was collected, hormonal concentrations were measured, and the secretion rates of iT4, iCT, and iPTH were calculated from the plasma concentration and the flow rate of thyroid venous plasma in four rats, as described previously [6]. Briefly, a thin polyethylene catheter was inserted into one of the four branches of the thyroid veins while the three other branches were ligated. During each experiment, 13–17 consecutive thyroid venous blood samples, consisting of approximately 250 μl per sample taking for 6–14 min, were collected for measurement of the three different hormones. The blood loss was compensated for by infusing 4% Ficoll PM70 in heparinized bicarbonate buffer. ELISA kits for T4 (general free T4 ELISA Kit, Cloud-Clone Corp, CEA185Ge, Katy, USA), CT (rat CT ELISA kit, MBS703165, MyBioSource, San Diego, CA, USA), and PTH (Rat Intact PTH ELISA Kit, Immutopics, San Clemente, CA, USA) were used to measure the concentrations of iT4, iCT, and iPTH in thyroid venous plasma, respectively.

### Transection of the SLN

**Fig. 2:**
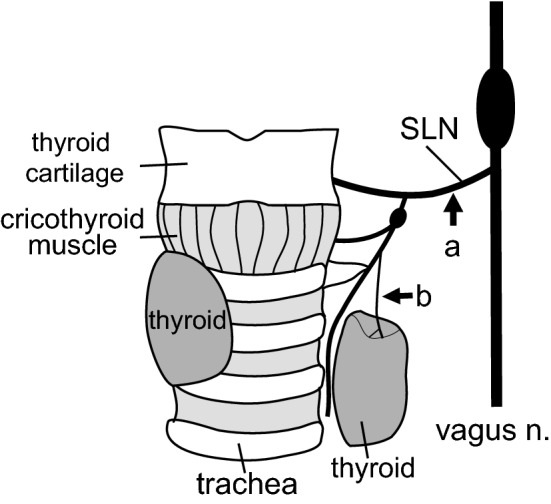
Schematic diagram of the anatomical arrangement of the SLN main trunk (**a**) and its branch, the thyroid nerve (**b**), on the left side in one rat, as seen from a ventral approach. The left thyroid lobe is separated from the right lobe and the trachea to show the thyroid nerve running on the medial surface of the thyroid

In blood sampling experiments, we examined the effect of pharyngeal stimulation on intact SLNs. Then we cut the SLNs to examine their contribution in pharyngeal stimulation-induced responses. The main trunks of the SLN (a, Fig. 2) were cut bilaterally at approximately 4–6 mm lateral to the thyroid gland in four rats.

### Recording of afferent nerve activity from the SLN

Afferent nerve discharges were recorded from the SLN innervating the pharynx in five rats. After the cervical skin was cut at midline in a supine position, the sternohyoid muscles were removed. Then a main trunk of the SLN (a, Fig. 2), either on the left or right side, was dissected and cut as close as possible to the nodose ganglion. The nerve dissected was covered with warm liquid paraffin, and afferent mass discharges were recorded from the cut peripheral end of the SLN, led through a bipolar platinum-iridium wire electrode and amplified by a preamplifier (S-0476, Nihon Kohden) using a 0.01-s time constant. Gallamine-triethiodide (20 mg/kg, i.v.) was used to avoid contamination of electrical activity of skeletal muscles. Discharge activity was constantly monitored visually on an oscilloscope, and audibly with a speaker, for any artifacts’ contamination during recordings. In three rats, we split the nerve using fine-tip forceps to record multiple or single unitary nerve activity, as described previously [22].

### Recording of efferent nerve activity from thyroid branches of the parasympathetic and sympathetic nerves

The efferent discharges were recorded from the central cut end of the thyroid branches of the parasympathetic and sympathetic nerves, using a bipolar platinum-iridium wire electrode and an amplifier, with gallamine, as described above for afferent nerve recording.

The thyroid nerve, a branch of external SLN, comes from the thyroid ganglion [23], runs along thyroid artery and vein, and then penetrates the sheath at the medial surface of the thyroid gland. The thyroid nerve was dissected and cut as close as possible to the thyroid gland (b, Fig. 2) in six rats. CSTs were cut bilaterally to avoid contamination of sympathetic efferent nerve activity. We confirmed the activity did arise from the postganglionic parasympathetic nerve, as it disappeared by administration of a ganglionic blocker (hexamethonium, 20 mg/kg, i.v.) at the end of recording in five of the six rats.

In the other three rats, the sympathetic nerve was dissected from a postganglionic branch coming from the cervical sympathetic ganglia and running toward the thyroid gland. The nerve branch was cut near the superior thyroid artery, about 3–5 mm distal to the cervical sympathetic ganglia. We confirmed the activity was derived from the sympathetic nerve, as it almost disappeared by transection of the ipsilateral cervical sympathetic trunk at the end of recording in each rat.

### Data analysis

The analog signals of blood pressure and nerve activity were digitized (Micro1401, Cambridge Electronic Design, UK) and analyzed using software (Spike 2, Cambridge Electronic Design, UK). Results are given as the mean ± standard error (SE), and data were evaluated statistically using the Student’s (unpaired) *t* test or paired *t* tests (Prism 5; GraphPad Software Inc., La Jolla, CA, USA). The statistical significance level was set at 5%.

## Results

### Changes in iT4, iCT, and iPTH secretion in response to pharyngeal stimulation

#### Resting state

The basal secretion rate of iT4, iCT, and iPTH, calculated from the concentration in thyroid venous plasma and the thyroid venous plasma flow rate under the resting condition before applying any stimulation in the four rats, was in the range of 0.31–0.50 pg/min, 0.50–0.95 pg/min, and 1.20–78.9 pg/min, respectively. Each value was stable in the absence of stimulation throughout approximately 3 h of continuous blood sampling in all four rats. Hematocrit values ranged from 41 to 56% at the first sampling and decreased to 30–49% at the last sampling, remaining above 30% in all samples measured. Plasma flow rate ranged from 12 to 13 μl/min at the first sampling and was 12–16 μl/min at the last sampling, showing no significant difference.

#### Responses to pharyngeal stimulation with intact SLNs

**Fig. 3:**
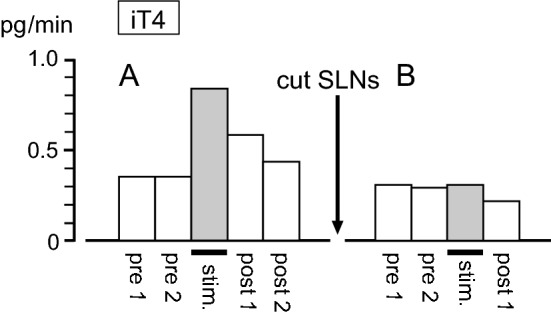
Representative example of changes in iT4 secretion in thyroid venous blood plasma induced by pharyngeal stimulation in one rat, before (**a**) and after (**b**) transection of the SLNs

**Fig. 4:**
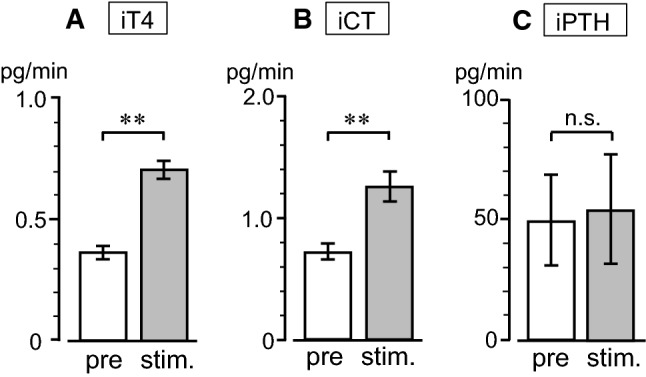
Summary of changes in the secretion rates of iT4 (**a**), iCT (**b**), and iPTH (**c**) in thyroid venous blood plasma in response to pharyngeal stimulation. Each column and vertical bar indicates mean ± SE (*n* = 6 in four rats). **p < 0.01; significantly different from prestimulus control values, using a paired *t* test

Mechanical stimulation was applied to the pharynx for a duration of 6–9 min. Figure 3a shows a representative example of iT4 secretion rate in response to pharyngeal stimulation in a rat with intact SLNs. The iT4 secretion rate doubled during the stimulation and decreased toward prestimulus control level after the stimulation. A similar response was observed in all six trials in the four rats tested. The iT4 secretion rate was 0.37 ± 0.03 (mean ± SE) pg/min before stimulation and significantly (*p* < 0.01, by paired *t* test) increased to 0.70 ± 0.04 pg/min during stimulation (Fig. 4a). The pharyngeal stimulation also produced an increase in iCT secretion rate in thyroid venous plasma during stimulation in all six trials in the four rats tested. Pharyngeal stimulation significantly (*p* < 0.01, by paired *t* test) increased iCT secretion rate from the prestimulus level of 0.72 ± 0.06 pg/min to 1.25 ± 0.12 pg/min during stimulation (Fig. 4b). When the same stimulation was applied in the same animal tested a second time, the iT4 and iCT responses were reproduced in the two rats tested. In contrast, the pharyngeal stimulation was ineffective in producing any consistent changes in iPTH secretion (Fig. 4c).

The plasma flow rate and mean arterial blood pressure, simultaneously monitored, increased significantly during pharyngeal stimulation (from 13.3 ± 0.4 to 24.3 ± 1.7 µl/min; and from 81.3 ± 4.7 to 101.2 ± 4.0 mmHg, respectively, *p* < 0.01, by paired *t* test). The increase reached 83% of the prestimulus control value for plasma flow and 24% for blood pressure. These responses, including secretion of iT4, iCT, and iPTH, flow rate, and blood pressure, induced by pharyngeal stimulation were all similar to those reported during electrical stimulation of the intact SLNs [6].

#### Responses to pharyngeal stimulation after SLNs’ transection

**Fig. 5:**
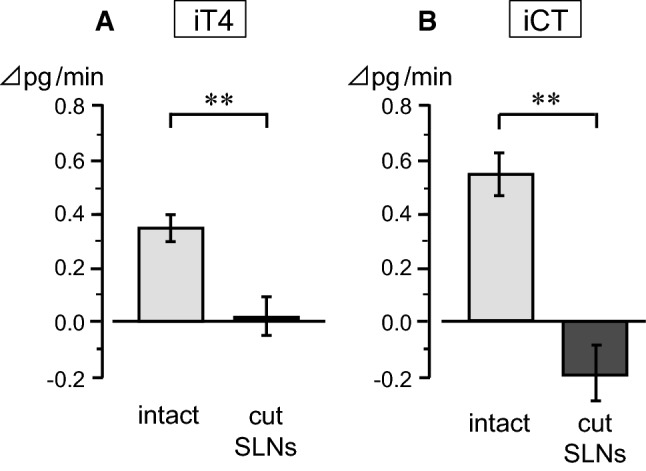
Effect of SLNs’ transection on iT4 (**a**) and iCT (**b**) secretion responses to pharyngeal stimulation. The responses are expressed as changes from prestimulus control values. Each column and vertical bar indicates a mean ± SE (*n* = 6 for intact, *n* = 5 for cut SLNs, in four rats). **p < 0.01; significantly different from control responses with intact SLNs, using unpaired *t* tests

The effect of pharyngeal stimulation on iT4 and iCT secretion after transection of the entire trunks of bilateral SLNs, in comparison with intact SLNs, was examined in four rats. This transection experiment aimed to define the role of the SLN in mediating the pharyngeal stimulus-induced increases in iT4 and iCT secretion. As shown in an example measure (Fig. 3b), SLNs transection scarcely affected the basal level of secretion. The basal secretion of iT4 or iCT was not different after SLNs’ transection, in accordance with our previous study [6]. The pharyngeal stimulation did not affect the secretion rate of iT4 (Fig. 3b, Fig. 5a) and iCT (Fig. 5b) after SLNs’ transection, in contrast to the marked increase observed in the control condition with intact SLNs (five trials in four rats). SLNs transection significantly reduced the changes in iT4 and iCT secretion during pharyngeal stimulation (*p* < 0.01, by Student’s *t* test) compared to those with intact SLNs (Fig. 5).

### Afferent discharges of the SLN innervating the pharynx in response to pharyngeal stimulation

**Fig. 6:**
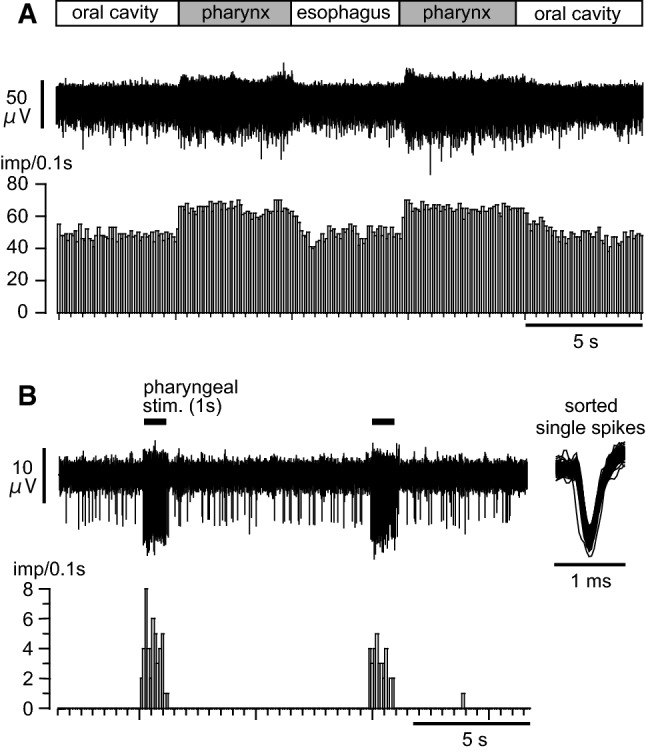
Afferent discharges of the SLN following pharyngeal stimulation. **a** Example of a mass afferent nerve recording. The lower histogram shows nerve activity counted consecutively every 0.1 s. **b** Example of a multiple unitary afferent nerve fiber recording. Horizontal lines indicate the period of stimulation. The right inset shows superimposed action potentials responding to pharyngeal stimulation after spike sorting. The lower histogram shows pulse count of the sorted nerve activity

Afferent mass activities in the SLN innervating the pharynx were recorded from the peripheral cut end segment of the SLN. During recording of afferent mass discharges of the SLN, we initially applied a pharyngeal stimulation for 5 s and compared the response to an esophageal stimulation for 5 s. Figure 6a shows a typical recording demonstrating that the discharges of the SLN afferent nerves increased when the balloon was in the pharyngeal cavity for 5 s. Of note, the activity started to increase within 0.2 s after the onset of pharyngeal stimulation, remained increased during the 5-s stimulation, and recovered within a few seconds after the stimulation. The SLN afferent activity did not change when the balloon was located either in the oral cavity or in the esophageal cavity. The location dependency results as shown in Fig. 6a were observed in three rats.

Figure 6b shows an example of multiple unitary afferent recording in response to a 1-s pharyngeal stimulation repeated every 10 s, corresponding to the stimulation used for blood sampling experiments. The largest-amplitude unitary spike activity occurred selectively during pharyngeal stimulation. In this unit, there were no spontaneous discharges in the resting state before pharyngeal stimulation. The peak activity of the unit during pharyngeal stimulation (for 1 s, repeated three times) was 8 imp/0.1 s. We observed similar responses in all six units recorded in three rats. The peak activity of the six units during pharyngeal stimulation ranged from 5 to 13 imp/0.1 s (8.0 ± 1.4 imp/0.1 s).

### Changes in thyroid autonomic efferent nerve activity in response to pharyngeal stimulation

**Fig. 7:**
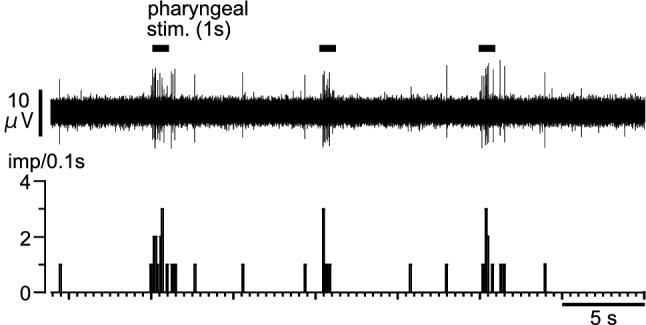
Effects of pharyngeal stimulation on thyroid parasympathetic SLN efferent nerve activity. Upper: Sample recording of thyroid efferent nerve responses following repeated pharyngeal stimulation in a rat. Lower: Histogram showing pulse count of the activity. Horizontal lines indicate the period of stimulation

The thyroid branch of parasympathetic SLN efferent nerves scarcely had spontaneous discharges at rest. However, pharyngeal stimulation (for 1 s, repeated every 10 s) evoked burst discharges during stimulation, as shown in a representative recording (Fig. 7). Similar responses were observed in all six rats tested. The averaged peak activity in six rats during pharyngeal stimulation (for 1 s, repeated three times) were 7.8 ± 1.9 imp/0.1 s. In contrast, the thyroid branch of sympathetic efferent nerves had spontaneous discharges at rest; these were either unchanged (*n* = 2) or only marginally increased (*n* = 1) during pharyngeal stimulation (tested in three rats).

## Discussion

In the present study, we demonstrated for the first time in anesthetized rats that a pharyngeal mechanical stimulation increased both iT4 and iCT secretion from the thyroid gland and generated burst discharges of thyroid parasympathetic SLN efferent nerves. As the iT4 and iCT secretions increased during electrical stimulation of the SLN efferent nerves [6], the pharyngeal stimulation-induced increases in iT4 and iCT secretion should be attributed to the reflex increase in parasympathetic SLN efferent nerve activity. Furthermore, since the pharyngeal stimulation-induced responses of iT4 and iCT secretion were totally abolished after bilateral transection of the SLNs, it is evident that the hormonal responses were produced by neurogenic reflexes via the SLN.

### Afferent pathway

Based on the responses induced by electrical stimulation of intact or cut SLNs, we have hypothesized that the excitation of myelinated SLN afferents promotes hormonal secretion from the thyroid gland via reflex activation of efferent nerve fibers in the SLN [6]. The present results provide evidence supporting this hypothesis and clarify that mechanical pharyngeal stimulation, such as during food swallowing, triggers such a reflex. Myelinated afferent units of rats’ SLNs respond to the positioning and passive movement of the thyroepiglottic joint or probing stimulation to the pharyngeal mucosa and surrounding tissues [24, 25]. Myelinated SLN afferents can discharge at frequencies of 20–80 Hz in response to mechanical stimuli [26]. The present facilitation of the iT4 and iCT secretion was elicited by a gentle mechanical stimulation applied to the pharynx by moving a soft deflated balloon. We confirmed that the same pharyngeal stimulation by a balloon activated the SLN afferent nerve. However, our results do not exclude the additional involvement of afferent nerves other than the SLN, such as the glossopharyngeal nerve, which shares innervation of pharyngeal mucosa with the SLN [18, 27, 28].

### Efferent pathway

The present responses during pharyngeal stimulation, i.e., increased iT4 and iCT secretions and unchanged iPTH secretions, were similar to those during the electrical stimulation of the SLN efferent, and different from those during the electrical stimulation of the sympathetic CST efferent (decreased iT4 and iCT secretion and increased iPTH secretion) that we reported previously [6]. Basal sympathetic activity appears to tonically suppress hormonal secretion from the thyroid gland [6]. Therefore, the increased iT4 and iCT secretion during pharyngeal stimulation could also be produced by a decrease in basal sympathetic nerve activity. However, the decrease in thyroid sympathetic nerve activity was not observed, whereas an apparent increase in thyroid parasympathetic nerve activity was observed during the pharyngeal stimulation in this study. Therefore, the increase in the parasympathetic nerve activity represents the main efferent pathway of reflex increases in iT4 and iCT by pharyngeal stimulation.

### Possible reflex center

**Fig. 8:**
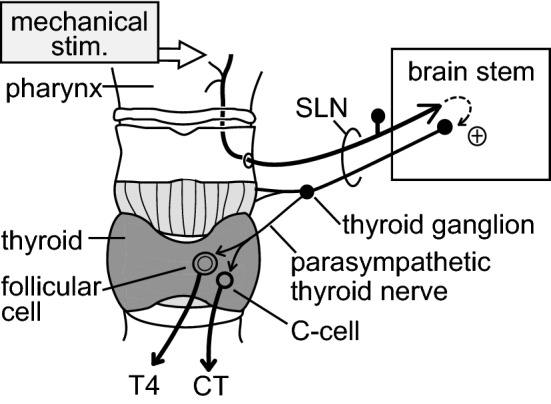
Schematic diagram of the proposed reflex pathway. A natural mechanical stimulation applied to the pharynx produces the secretion of T4 and CT from the thyroid gland as a consequence of the segmental brainstem reflex via the SLN

The mechanical stimulation of the pharynx activated afferent nerves in the SLN and parasympathetic efferent nerves in the thyroid branch of the SLN. Thus, we may conclude that the central connections between pharyngeal mechanoreceptive afferents and thyroid parasympathetic preganglionic neurons are essential. SLN afferent fibers project to the ipsilateral region of the nucleus of the solitary tract in the brainstem [16, 29], whereas thyroid parasympathetic efferent neurons originate from the rostral part of the dorsal motor nucleus of the vagus [30]. Oral-pharyngeal and esophageal stimulation by sham feeding induced c-fos expression in these nuclei [31]. We predict that excitation of the mechanoreceptive afferent nerve fibers in the SLN by pharyngeal stimulation produces a reflex increase in thyroid parasympathetic efferent nerve activity, probably via these brainstem nuclei, leading to an increase in hormonal secretion from the thyroid (Fig. 8).

### Physiological significance

The plasma iT4 and iCT levels in systemic blood increase rapidly after a meal in a conscious state in humans [32] and animals [33–35]. In particular, the plasma iT4 level increased by 37% after a meal in young growing pigs [35], and the plasma iCT level doubled after breakfast in humans [32]. Increased secretion of iT4 and iCT after a meal was observed independently of changes in humoral regulatory factors, such as thyroid stimulating hormone and calcium, and the mechanisms underlying these responses were undetermined. The mechanism of pharyngeal stimulation-induced increases in iT4 and iCT secretions from the thyroid, shown in our study, partially explain the facilitation of iT4 and iCT secretions in response to food intake.

## Conclusions

In conclusion, the promotion of iT4 and iCT secretion induced by mechanical stimulation of the pharyngeal area is the consequence of a segmentally organized reflex response. Afferent arcs include SLN mechanoreceptive afferent nerve branches innervating the pharyngeal mucosa and surrounding muscles and epiglottis. The efferent arc is represented by the SLN autonomic (parasympathetic) efferent nerve innervating the thyroid gland (Fig. 8). However, other natural stimuli, such as the chemical stimulation of the epiglottis and larynx [36, 37] or vocal stimulation [38], which are known to activate SLN myelinated afferents, might also promote the secretory function of the thyroid. Future studies are needed to clarify these possibilities.
